# The Application of GeneXpert MTB/RIF for Smear-Negative TB Diagnosis as a Fee-Paying Service at a South Asian General Hospital

**DOI:** 10.1155/2015/102430

**Published:** 2015-04-08

**Authors:** Poojan Shrestha, Amit Arjyal, Maxine Caws, Krishna Govinda Prajapati, Abhilasha Karkey, Sabina Dongol, Saruna Pathak, Shanti Prajapati, Buddha Basnyat

**Affiliations:** ^1^Oxford University Clinical Research Unit, Patan Hospital, Patan Academy of Health Sciences, P.O. Box 252, Kathmandu, Nepal; ^2^Department of Clinical Sciences, Liverpool School of Tropical Medicine, Pembroke Place, Liverpool L3 5QA, UK; ^3^Oxford University Clinical Research Unit, Hospital for Tropical Diseases, Wellcome Trust Major Overseas Programme, 764 Vo Van Kiet, District 5, Ho Chi Minh City, Vietnam; ^4^Department of Microbiology and Immunology, Patan Hospital, Patan Academy of Health Sciences, P.O. Box 252, Kathmandu, Nepal

## Abstract

The GeneXpert MTB/RIF assay (Xpert) is a novel automated diagnostic tool for tuberculosis but its optimal placement in the healthcare system has not been determined. The objective of this study was to determine the possibility of additional case detection for pulmonary tuberculosis (PTB) by offering Xpert to smear-negative patients in a low-HIV burden setting with no* Mycobacterium tuberculosis *(*M.tb.*) culture facilities. Patients routinely presenting with symptoms suggestive of PTB with negative smears were offered single Xpert test on a fee-paying basis. Data were retrospectively reviewed to determine case detection in patients tested from February to December 2013. Symptoms associated with a positive test were analysed to determine if refinement of clinical criteria would reduce unnecessary testing. 258 smear-negative patients were included and* M.tb. *was detected in 55 (21.32%, *n* = 55/258). Using standard clinical assessment for selection, testing 5 patients detected one case of smear-negative PTB. These results demonstrate that fee-paying Xpert service in low-income setting can increase TB case confirmation substantially and further systematic studies of health economic implications should be conducted to determine optimal implementation models to increase access to Xpert in low- and middle-income countries.

## 1. Introduction

Tuberculosis (TB) continues to be a major public health problem, with 8 million cases and 1.3 million deaths each year [1]. One of the key challenges for TB control is to increase case detection and early treatment, thereby interrupting transmission chains and reducing individual morbidity. The most widely used test for TB, sputum smear microscopy, has a sensitivity of only 50% of active cases which contributes to delayed diagnosis resulting in continued transmission [2, 3]. In an advanced diagnostic setting, facilities such as culture, drug susceptibility testing (DST), and commercial molecular diagnostics may be available, but these facilities are lacking in most hospitals in high burden countries. Sputum smears with chest X-ray (CXR), where available, are the tests routinely applied for TB diagnosis. It is crucial to implement improved diagnostics in these endemic settings if we are to reach the targets of case detection, reduction in mortality, and prevalence of the disease [4, 5].

Derivations from case burden estimates suggest that only around 70% of active TB cases are diagnosed using current strategies [1]. The development and implementation of the Xpert assay (Cepheid, Sunnyvale, CA, USA), which has high sensitivity in detection of smear-negative TB, has raised hopes of increased case detection in low- and middle-income countries (LMICs). Xpert is the only fully automated real-time DNA-based test which can detect both TB and rifampicin resistance (RR) [6, 7]. The test is based on a heminested PCR test which detects the presence of* Mycobacterium tuberculosis* complex bacilli [8]. The PCR target, an 81-base-pair region of the* rpoB* gene, is the rifampicin resistance determining region (RRDR) [6, 9]. The reactions take place in a single-use cartridge making it easy to operate, without cross contamination, and giving a result in as little as 2 hours and thus potentially decreasing default due to delayed diagnosis [7, 10].

In the most recent meta-analysis, Xpert as an initial replacement for smear microscopy showed a pooled sensitivity of 89% (95% credible interval CrI 85% to 92%) and pooled specificity of 99% (95% CrI 98% to 99%), and as an add-on test following a negative smear microscopy, pooled sensitivity and specificity were 67% (95% CrI 60% to 74%) and 99% (95% CrI 98% to 99%), respectively [11]. For RR detection, it achieved a pooled sensitivity of 95% (95% CrI 90% to 97%) and a pooled specificity of 98% (95% CrI 97% to 99%) [11]. The test was first endorsed by WHO in 2010 [11, 12] and revised recommendations in 2013 included a recommendation that Xpert be used in all suspected TB cases, with the acknowledgement that this has substantial resource limitations [13].

However, despite a substantial negotiated price reduction on the test for LMICs, the 10 USD per cartridge remains expensive for low-income countries which often have per capita healthcare expenditure of <30 USD [14]. There have been limited reports of Xpert implementation on a fee-paying basis in such settings, with the majority of projects implementing free at point of care, a model which is dependent on sustained aid funding. Although the test cost is high to the patient, a confirmed diagnosis can avert often lengthy differential investigations and exploratory treatment which may cost substantially more. We therefore examined the impact on case detection of implementing Xpert as an optional fee-paying service to patients with suspected smear-negative TB.

In Nepal, the estimated incidence of TB is 163 per 100,000 with a prevalence rate of 241 per 100,000 population. In 2012-13, the Nepal Tuberculosis Programme (NTP) registered 17,788 sputum smear-positive cases and 8,367 sputum smear-negative cases [15]. In common with many LMICs, many pulmonary TB cases treated in the private sector go unreported and therefore there is likely to be discrepancy between official figures and the true numbers being diagnosed and treated. Although multidrug resistant tuberculosis (MDR TB) rates remain relatively low, at 2.2% in new and 17.2% among retreatment cases, only 262, one-quarter of the estimated MDR cases, were treated via the national programme in 2012-13 [15]. The positive predictive value of rifampicin resistance detection using Xpert in an unselected patient population will be low and confirmation of MDR is necessary before MDR treatment is initiated.

A recent Cochrane study concluded that more research would be helpful in evaluating the use of Xpert in TB programmes in high TB burden settings [11].

We report here the experience of implementing Xpert at a general hospital in Kathmandu, Nepal, and determine the number needed to test in order to detect one active case of smear-negative TB.

## 2. Materials and Methods

Patan Hospital is a 450-bed government general hospital providing emergency and elective outpatient and inpatient services, located in the Lalitpur area of the Kathmandu valley. The laboratory services are limited with acid-fast bacilli smear available but no provision for* M.tb.* culture.

From February 18, 2013, an Xpert machine was made available for testing. Clinical practitioners both from within and outside the hospital were invited to refer patients with symptoms consistent with TB and three negative sputum smears to testing by Xpert. Patients offered the test were informed about the test evaluation and asked for consent to collect baseline data on demographics and symptoms at presentation. This study was approved by the Institutional Review Board of Patan Hospital.

Patients with 3 negative Ziehl-Neelsen (ZN) sputum smears were referred for Xpert testing at the treating clinician's discretion. A new sputum sample was collected for Xpert testing. Data was collected on history of persistent cough (≥2 weeks), fever, drenching night sweats, weight loss (>1.5 kg in a month), loss of appetite, malaise, and shortness of breath or chest pain.

CXR reports were collected where available. For further analysis, CXR features were classified by the study investigators according to radiologist's report as upper lobe infiltrates, pleural effusion, diffuse infiltrates, cavitary lesions, other infiltrates, consolidation, other abnormalities, and normal. CXR reported as patchy infiltrates, bilateral infiltrates, and infiltrates without a specified location was classified as “other infiltrates.” “Other abnormalities” comprised of pneumothorax, hyperinflation, lung abscess, mediastinal opacity, nodular opacity, mass lesion, and pleural thickening.

At Patan Hospital, the acid-fast bacilli microscopy is performed by experienced laboratory personnel following the guidelines set by the NTP and is monitored by the NTP external quality assessment (EQA) scheme. The sputum sample for the Xpert was processed according to manufacturer's guidelines. The patients were charged NPR 2000 (~20 USD) per test. This cost included freight charges (~$3 per cartridge), staffing, annual machine calibration ($450 per year), and annual maintenance charge (~$2,700 per year). The HIV status was reported by the patient but no confirmatory HIV testing was performed. Data on the treatment outcome was not collected.

We further examined whether restricting the tested population would miss cases of TB or improve efficiency of testing and reduce unnecessary patient charges. We determined the number needed to test if the tested population was restricted to (1) those with 2 or more symptoms consistent with TB (fever, night sweats, persistent cough (≥2 weeks), weight loss (≥1.5 kg in a month), malaise, shortness of breath, or chest pain) or (2) those with CXR abnormalities and 2 or more symptoms or (3) those with only the CXR abnormalities found in the Xpert positive group.

Categorical variables were compared between patient groups testing positive and negative by Xpert using Fisher's exact test with a *P* value of ≤0.05 considered as significant.

## 3. Results 

Between February 18, 2013, and December 30, 2013, 258 smear-negative patients were tested by Xpert on sputum. Eighty-nine patients were referred from other hospitals throughout the Kathmandu valley, while 169 were from Patan Hospital clinics. Data were not systematically collected on patients who were smear-negative but not offered the test during the study period but no patients declined the test when offered at Patan Hospital. No patient declined consent for data collection and we therefore included all Xpert tested patients in the analysis.

During the study period, there were 2,222 new patients tested by smear microscopy at Patan Hospital. Of these, 1,070 had 3 negative sputum smears and 196 had at least one positive sputum smear. The remaining 956 patients did not complete 3 sputum smears. Therefore, 169/1070 (15.8%) of diagnostic subjects were offered an Xpert test. In the majority of cases, this was due to an alternative diagnosis being reached or symptom resolution/improvement during the smear and CXR diagnostic process, but this was not systematically evaluated.

Xpert was positive for* M.tb.* in 55 (*n* = 55/258, 21.3%) patients. Therefore, 4.7 patients needed to be tested to detect one active TB case. All patients with a positive Xpert test were referred for free treatment in the appropriate NTP DOTS facility.

A third of patients (*n* = 93/258, 37.98%) was female and the median age was 52 (interquartile range IQR 33–68). Four children were included (13, 15, and two 17 years of age). There were 2 patients (*n* = 2/258, 0.8%) with a positive result for rifampicin resistant TB by the Xpert test. The first patient was referred for sputum culture and phenotypic drug susceptibility testing at a tertiary centre (GENETUP, Nepal). The second rifampicin resistant patient was untraceable for follow-up by the phone number provided. No patient reported known HIV infection.

Table 1 shows the clinical features of the patients. The only clinical feature to show a statistically significant difference between the groups was shortness of breath, which was more common among Xpert negative patients (56.6 versus 72.2%, *P* = 0.04). As this finding nevertheless occurred in half of Xpert positive patients, it was not appropriate for refining the group of patients tested.

CXR was available for 187 patients (Table 2). Thirteen patients (*n* = 13/187, 6.9%) had no abnormality detected on CXR. Upper lobe infiltrates (*P* = 0.03) and cavitary lesions (*P* = 0.03) were more common among the Xpert positive group. Consolidation (*P* = 0.04) was not evident among the Xpert positive group while it was reported in 9.4% of Xpert negative patients.

No clinical questionnaire was available for 7 patients, leaving 251 patients in the symptom analysis (Table 3). If the Xpert testing was restricted to patients with 2 or more symptoms, the number needed to test was not altered and remained at 4.6 (*n* = 50/232, 21.6% positive). Three cases of TB would have been missed.

187 patients had an available questionnaire and CXR report. If CXR abnormalities and 2 clinical symptoms were required for Xpert testing, the number needed to test was 4.5 (*n* = 36/163, 22.1%). Three cases of TB would have been missed.

187 patients had CXR available. If testing were restricted to only those with an abnormal CXR showing at least one of the four characteristics, upper lobe infiltrates, cavitary lesions, pleural effusion, or infiltrates, then the number needed to test was 4 (*n* = 38/152 = 25%). 1 case would have been missed.

## 4. Discussion

This report demonstrates that application of Xpert testing as a fee-paying service in a general hospital can substantially increase the yield of confirmed TB cases. Five patients need to be tested to detect one TB case by applying simple routine criteria to select smear-negative TB suspects. During the same time period, there were 196 smear-positive TB cases at Patan Hospital and the implementation of Xpert therefore resulted in a 28.1% (*n* = 55/196) increase in confirmed TB cases.

We assumed that the specificity of Xpert is high, as every study performed to date and meta-analysis has indicated consistently high specificity of 99% [10, 11, 16]. Therefore, although there is no culture confirmation for comparison, this was not an evaluation of diagnostic accuracy, which has been comprehensively reported elsewhere and it can be reasonably assumed that these cases are genuine, and the rate of false positive diagnosis is unlikely to exceed that of culture.

In areas of high TB prevalence such as Nepal, the majority of suspected TB cases are assessed by sputum smear microscopy and, where available, by CXR. Patients are often placed onto TB treatment on the basis of persistent cough or abnormal CXR alone. Modelling studies have indicated that Xpert scale-up may not increase the overall number of cases initiated on treatment due to these pragmatic empirical treatment practices. Instead, Xpert will divert treatment away from “false cases” to “true” smear-negative TB cases, thereby increasing the accuracy of treatment and cost-effectiveness, while reducing the burdens of toxicity and opportunity cost of treatment in patients who do not in fact have TB [17, 18]. The ability of Xpert to rapidly confirm TB in smear-negative cases offers the possibility of improving early TB case detection, but due to costs, recommendations for high burden, low-resource settings have so far focused on applying Xpert to HIV-infected individuals (where the sensitivity of smear is especially low and the complications of missed diagnosis are more severe) and patients with risk factors for MDR TB. Although WHO recommends applying the test to all smear-negative cases, the recommendation comes with the caveat that this is not financially feasible in most settings [13]. The low positive predictive value of Xpert for rifampicin resistance in a low MDR prevalence test population must also be considered. There has been considerable debate around the possibility of providing Xpert on a fee-paying basis to patients in low-income, high burden settings [19]. This study demonstrates that both uptake and case detection can be high in a targeted approach but further studies should be performed to determine the health economic impacts of such application, both on the individual patients including the opportunity costs of alternative health investigations and on household finances of testing costs and on hospital resource allocation.

The impact of delayed TB detection is threefold: firstly, morbidity to the individual is increased and in many cases will persist beyond the disease episode in severe permanent lung damage; secondly, undetected smear-negative TB will become progressively more infectious and transmit within the community; and finally the economic impact on the household is magnified by repeated visits to healthcare facilities, differential diagnostic testing and treatments, and loss of earnings due to healthcare seeking and morbidity.

The Xpert assay has also increased early detection of MDR-TB, particularly when applied to high-risk groups in accordance with WHO recommendations. Prior to application of this assay, patients at high risk for MDR TB would have to be referred to a tertiary setting and wait 6 to 8 weeks for results of phenotypic drug susceptibility testing resulting in high loss to follow-up and delays in treatment initiation. The line probe assays for MDR diagnosis have also largely been limited to tertiary centres in LMICs. In an unselected patient group without MDR risk factors and a low background prevalence of MDR TB, the positive predictive value of Xpert for rifampicin resistance is low and the result should be confirmed by a second test. However, early identification of possible MDR cases is key to reducing community transmission and reducing the incidence of MDR TB. The first patient positive for rifampicin resistant TB by Xpert in this study was referred to a tertiary centre for confirmatory testing by phenotypic DST according to the national guidelines. The phenotypic DST showed the patient was infected with TB resistant to isoniazid, rifampicin, streptomycin, and ethambutol. Without Xpert testing, this patient would have received a first line treatment regimen for a minimum of 5 months before being tested by phenotypic DST. The loss to follow-up of the second case despite the use of a rapid test demonstrates the problems associated with TB control beyond the need for accurate tests. This case could not be confirmed as a true positive for MDR and may not have received any TB treatment.

It is not clear how many of the patients testing positive for* M.tb.* by Xpert in this study would have been started on TB treatment based on the judgement of the treating clinician if Xpert testing was not available [20]. In the previous year (2011-2012) at Patan Hospital, 21 patients were empirically treated for TB, approximately one-third of those detected by Xpert during the study period (1.75/months versus 5.65/months, resp.). It is possible that the availability of Xpert increased the recognition of TB symptoms, although we can claim that in this endemic setting TB awareness is consistently high among clinicians. Conversely, it is possible that a negative Xpert test may have discouraged treatment in some cases which would otherwise have been treated, although clinicians were informed that a negative Xpert test does not exclude a TB diagnosis. Patients with a strong possibility of pulmonary TB despite a negative Xpert test were referred for further investigations including culture at a tertiary centre. The rate of smear positivity at Patan is 9.28% which is consistent with the results in other areas of similar endemicity; it is therefore unlikely that the Xpert diagnosis was inflated due to low smear microscopy confirmation [21–23].

The cost to the patients of the assay was 20 USD (which is equivalent to the price of eight CXRs in our hospital), including the full cost to the hospital of performing the assay and maintaining the service. Despite this, no clinician reported patients refusing the test for financial reasons, although this was not systematically evaluated. Studies of the patient acceptability of testing should be performed to determine the relative cost benefits for the patients of receiving a fee-paying Xpert test versus the traditional diagnostic route.

Scale-up of Xpert testing is underway in Nepal but funding is not available to provide Xpert testing free to all patients with suspected smear-negative TB and is unlikely to be so in the near to medium term. This is also true in other LMICs. In India, it was calculated that providing Xpert to all smear-negative TB suspects would consume the entire national healthcare budget [17].

Our study is consistent with other studies which have suggested the benefit of Xpert in smear-negative patients in developing countries [11, 24, 25]. The majority of these studies have been carried out in Africa where there is a substantially higher HIV burden than in South Asia. Three studies have been reported from low-HIV prevalence regions, including Peru and two hospitals (Hinduja Hospital and Christian Medical College Hospital) in India [8, 18, 25].

We examined the clinical features of groups testing positive and negative by Xpert to determine if further refinement of eligibility for testing criteria could guide the application of Xpert and reduce unnecessary testing and thereby costs to the patient. The number needed to test was reduced to 4 by restricting patients tested to only those with specific CXR criteria consistent with TB, while only a single case was missed. This strategy should be evaluated in a larger multicentre study to determine the optimal referral algorithm for Xpert testing in the general hospital setting.

By using only the classical broad definition of TB symptoms and CXR interpretation, the number needed to test for a single confirmed case of active TB is as low as five in this general hospital. Although smear microscopy, due to its simplicity, speed, and low cost, is used widely in low-resource settings, the low sensitivity precludes it from being an ideal test. The requirement for a rapid, simple TB diagnostic is evidenced by the widespread application of commercial serological tests which are inaccurate. These tests are widely provided at a cost to the patient and used to determine medical treatment. The government of India has recently banned the use of these tests following systematic evaluation by WHO [26]. Although the Xpert has several limitations, including requirement for stable electricity supply, limited temperature range, availability of maintenance, and bulky consumables, wider availability of the accurate Xpert assay may counter the use of these serological tests by providing a viable alternative to the patient and healthcare provider.

It is highly probable that a small number of cases of TB were missed in this study as Xpert does not have as high sensitivity as culture. There is a danger that clinicians will “exclude” a TB diagnosis on the basis of a negative Xpert test and it is important that education is carried out to ensure clinicians are aware of the test limitations prior to the test being implemented. However, it is not sustainable to implement TB culture facilities at general hospitals in South Asia and the long turnaround of results means loss to follow-up in the diagnostic pathway is high. This study was not an assessment of Xpert sensitivity and specificity, as this has been comprehensively evaluated in comparison with culture by others.

## 5. Conclusion

Our study has shown that by applying Xpert to test for smear-negative TB on a fee-paying basis in a general hospital in Nepal, five patients need to be tested to detect one case of active TB. Restriction of criteria for testing using CXR features can reduce the number needed to 4. Further research is needed evaluating the cost effectiveness, patient acceptability and impact on overall out-of-pocket expenditure of Xpert testing provided at a direct cost to the patient, to determine the optimal sustainable use of this technology while maximizing equality of access.

## Figures and Tables

**Table 1:** Comparison of clinical features of 251 patients with suspected smear negative TB testing positive and negative by Xpert.

Clinical features	Xpert positive	Xpert negative	*P* value
Cough	38 (71.7)	143 (72.2)	1
Fever	28 (52.8)	90 (45.5)	0.36
Night sweats	23 (43.4)	61 (30.8)	0.10
Loss of appetite	39 (73.6)	140 (70.7)	0.74
Weight loss	31 (58.5)	106 (53.5)	0.54
Malaise	42 (79.3)	164 (82.8)	0.55
Shortness of breath/chest pain	30 (56.6)	143 (72.2)	0.04

Total	53 (100)	198 (100)	

^∗^Clinical questionnaire was not available for 7 patients.

kg = kilogram.

**Table 2:** Comparison of chest X-ray features of 187 patients testing positive or negative by Xpert.

Chest X-ray findings	Xpert positive	Xpert negative	*P* value
Upper lobe infiltrates	12 (31.57)	22 (14.86)	0.03
Cavitary lesions	5 (10.52)	5 (4.05)	0.03
Pleural effusion	9 (29.68)	27 (18.24)	0.49
Diffuse infiltration	7 (18.42)	26 (17.56)	1
Consolidation	0	14 (9.45)	0.04
Others	0	11 (6.75)	0.12
Normal	1 (2.6)	12 (8.10)	0.30
Other infiltrates	6 (13.15)	24 (16.89)	1
Infiltrates not in upper lobes	5 (13.15)	21 (14.18)	1

Total	39 (100)	148 (100)	

^∗^Sum of the categories is greater than the total number of patients as one patient may be in more than one category.

**Table 3:** Number of patients detected as positive by GeneXpert using different inclusion criteria for testing.

Patients	Number of patients with information *n* (%)	Number of patients tested	Numbers of Xpert positive *n* (%)	Number needed to test	Numbers of patients not meeting the criteria	Xpert positive in group not meeting criteria *n* (%)
At least 2 symptoms^∗^	251 (93.7)	232	50 (21.6)	4.6	19	3 (15.8)
At least 2 symptoms + CXR^∗∗^	187 (72.5)	163	36 (22.1)	4.5	24	3 (12.5)
At least the 4 listed CXR abnormalities	187 (72.5)	152	38 (25.0)	4.0	35	1 (2.9)

^∗^7 patients had no available information of symptoms and were therefore excluded.

^∗∗^71 patients did not have information of CXR report and were therefore excluded.
